# Improved efficacy of allergen-specific immunotherapy by JAK inhibition in a murine model of allergic asthma

**DOI:** 10.1371/journal.pone.0178563

**Published:** 2017-06-01

**Authors:** Antonio Aguilar-Pimentel, Anke Graessel, Francesca Alessandrini, Helmut Fuchs, Valerie Gailus-Durner, Martin Hrabě de Angelis, Dennis Russkamp, Adam Chaker, Markus Ollert, Simon Blank, Jan Gutermuth, Carsten B. Schmidt-Weber

**Affiliations:** 1 German Mouse Clinic, Institute of Experimental Genetics, Helmholtz Center Munich, Neuherberg, Germany; 2 Center of Allergy and Environment (ZAUM), Technical University of Munich and Helmholtz Center Munich, Member of the German Center for Lung research (DZL), Munich, Germany; 3 Experimental Genetics, School of Life Science Weihenstephan, Technical University of Munich, Freising, Germany; 4 Department of Otolaryngology, Klinikum rechts der Isar, Technical University of Munich, Munich, Germany; 5 Department of Infection and Immunity, Luxembourg Institute of Health (LIH), Esch-sur-Alzette, Luxembourg; 6 Department of Dermatology and Allergy Center, Odense Research Center for Anaphylaxis, University of Southern Denmark, Odense, Denmark; 7 Department of Dermatology, Universitair Ziekenhuis Brussel, Vrije Universiteit Brussel, Brussels, Belgium; Centre National de la Recherche Scientifique, FRANCE

## Abstract

**Background:**

Allergen-specific immunotherapy (AIT) is the only curative treatment for type-1 allergies, but sometimes shows limited therapeutic response as well as local and systemic side effects. Limited control of local inflammation and patient symptoms hampers its widespread use in severe allergic asthma.

**Objective:**

Our aim was to evaluate whether AIT is more effective in suppression of local inflammation if performed under the umbrella of short-term non-specific immunomodulation using a small molecule inhibitor of JAK pathways.

**Methods:**

In C57BL/6J mice, a model of ovalbumin (OVA)-induced allergic airway inflammation and allergen-specific immunotherapy was combined with the administration of Tofacitinib (TOFA, a FDA-approved JAK inhibitor) from 48 hours prior to 48 hours after therapeutic OVA-injection. The effect of TOFA on human FOXP3^+^CD4^+^ T cells was studied *in vitro*.

**Results:**

AIT combined with short-term TOFA administration was significantly more effective in suppressing total cell and eosinophil infiltration into the lung, local cytokine production including IL-1β and CXCL1 and showed a trend for the reduction of IL-4, IL-13, TNF-α and IL-6 compared to AIT alone. Furthermore, TOFA co-administration significantly reduced systemic IL-6, IL-1β and OVA-specific IgE levels and induced IgG1 to the same extent as AIT alone. Additionally, TOFA enhanced the induction of human FOXP3^+^CD4^+^ T cells.

**Conclusions:**

This proof of concept study shows that JAK inhibition did not inhibit tolerance induction, but improved experimental AIT at the level of local inflammation. The improved control of local inflammation might extend the use of AIT in more severe conditions such as polyallergy, asthma and high-risk patients suffering from mastocytosis or anaphylaxis.

## Introduction

Allergen-specific immunotherapy (AIT) is the only curative therapy for allergic diseases, which is able to maintain its efficacy after discontinuation of treatment and which may significantly reduce allergic symptoms [1–3]. However, the therapeutic allergen exposure initially boosts allergic pathways, e.g. by increasing IgE secretion by B cells, success rates vary and patient compliance is low due to long treatment regimens. Moreover, the indication of AIT is mainly limited to allergic rhinitis and mild asthma, while this treatment is contra-indicated in severe allergic asthma [4] and of limited help in polyallergic rhinitis patients and atopic eczema [5]. Additionally, combining AIT with an anti-inflammatory therapy might facilitate the reduction of side effects and the acceleration of the titration phase.

Therefore, it is highly desirable that curative success of up to 95% in selected indications such as hymenoptera venom allergy [6] can also be translated into the tolerogenic vaccination of difficult-to-treat allergies affecting the respiratory tract, eyes or gut. Furthermore, it is of considerable interest to extend the application of AIT to atopic eczema and polyallergic patients, where this treatment is less efficient, because the local inflammatory process is not only causing side effects, but also impairs the induction of immune tolerance [7]. Hence, a vaccination-induced antigen presentation in the absence of inflammatory signals, induced by short-term JAK-pathway inhibition of inflammation, might facilitate the induction of immunological tolerance.

The mechanisms underlying the induction of allergen-specific immune tolerance are incompletely understood. To date, the induction of allergen-specific IgG4 and a change of the pro-allergic T cell phenotype are assumed to underlie long-lasting treatment benefits [8, 9]. Regulatory T cells (Tregs) were reported to be induced by AIT [10, 11] and may play a role in suppressing pro-allergic Th2 cells [12]. It is currently unclear how the induction of regulatory T cells can be facilitated in the context of antigen-specific vaccination. However, it appears that allergic inflammation, specifically IL-4-mediated inhibition of the *FOXP3* promoter [13], can directly inhibit the induction of regulatory T cells. Similarly, other pro-inflammatory mediators are counteracting Treg induction [14].

Consequently, the use of immunomodulating drugs may promote the induction of Tregs as long as they do not block signals that are necessary for the differentiation of Tregs. While Cyclosporine A inhibits the induction of Tregs [15] and Corticosteroids are only minimally promoting Tregs [16, 17], it was shown that Janus kinase (JAK) inhibitors preserve the Treg function [18]. JAKs are key players in cytokine-mediated activation of STATs (signal transducers and activators of transcription) and, therefore, of inflammatory processes [19]. The current study assessed the impact of short-term application of the FDA-approved JAK inhibitor Tofacitinib (TOFA) [20] on therapy outcome in a murine model of OVA (chicken ovalbumin)-specific immunotherapy. Moreover, the effect of TOFA on FOXP3 expression in human T cells was addressed. We hypothesized that the efficacy of AIT might be facilitated by the anti-inflammatory effects of TOFA administration for short periods of 5 days during the up-dosing phase, which are uncritical with respect to TOFA-mediated side effects [21].

## Methods

### Animals

Female C57BL/6J mice (Charles River, Sulzfeld, Germany) were housed under specific pathogen free conditions in GM500 cages, including individually ventilated caging systems (IVC System Green Line, Tecniplast, Buguggiate, Italy), which are operated with positive pressure. The mice are transferred to new cages with forceps in Laminar Flow Class II changing stations weekly; they are fed with an irradiated standard rodent high energy breeding diet (Altromin 1314, Altromin Spezialfutter GmbH & Co. KG, Lippe, Germany) and have access ad libitum to semi-demineralized filtered (0.2 mm) water. The light cycle is adjusted to a 12h/12h light/dark cycle; room temperature is regulated to 22 +/- 1°C and relative humidity to 55 +/- 5%. Husbandry conditions are adjusted to the experimental requirements in specified modules. Sentinels (outbred 8-week-old male SPF Swiss mice) are housed on a mixture (50:50) of new bedding material and a mixture of soiled bedding from all cages of this IVC rack and their health is monitored by on-site examination of certified laboratories according to the FELASA recommendations (http://www.felasa.org). All animal experiments were carried out in accordance with German legal guidelines and following the approval (approval number 55.2-1-54-2532-30-14) of the responsible animal welfare authorities and the ethics board of the district government of Upper Bavaria, Germany. Animals were monitored every day after *in vivo* intervention, otherwise weekly. The facility follows a surveillance protocol, which indicates early endpoints such as strong bodyweight loss, abnormal coat/behavior or issues related to infections. If any of these clinical signs were reached prior to the experimental endpoint, the animals were euthanized. Euthanasia was carried out by an overdose of Ketamin/Xylazine. Within the presented experiments, none of the animals died prior to the experimental endpoint.

### Allergen sensitization and AIT model

For allergic sensitization, mice were treated twice by intraperitoneal injection of 10 μg OVA (chicken ovalbumin; Sigma-Aldrich, Taufkirchen, Germany) and 0.5 mg aluminum hydroxide (imject^®^ Alum; 40 mg magnesium/40 mg alum per mL; Thermo Fischer Scientific, Waltham, MA USA) in 200 μl phosphate buffered saline (PBS) at day (D)-1 and D-7 as previously described [22, 23]. Subsequently, mice were challenged by inhalative exposure to OVA aerosol (1% in PBS) for 10 min once a day at D-49, 52 and 55. 24 h after the last challenge, blood samples were collected by puncturing the retro-orbital plexus (Li-heparin-coated tubes, KADE, Nümbrecht, Germany) under isoflurane anesthesia [22–24]. Blood samples were centrifuged (10 min, 5000 x g, 4°C) to separate cells and plasma. Animals were sacrificed to obtain bronchoalveolar lavage (BAL) samples as previously described [25].

For allergen-specific-immunotherapy (AIT), animals were treated by subcutaneous injection of 1 mg OVA at D-22, and 0.5 mg OVA at D-29. For treatment with Tofacitinib (TOFA), (Tofacitinib citrate; CP-690550; Selleckchem-Biozol Diagnostica Vertrieb GmbH, Eching, Germany, dissolved to 50 mg/ml in sterile DMSO) two doses per day were administered on D-20 to D-24, and D-27 to D-31 by oral gavage feeding (10.8 mg/kg in 200 μl water).

### Controls and treatment protocol

Within one experimental setup, statistically calculated to reach highly significant results, we treated and analyzed the following groups (n = 9–14) of mice:

Non-allergic: non-sensitized mice, exposed to OVA-aerosol only.

Allergic: mice sensitized with OVA and exposed to OVA-aerosol (challenge).

Allergic-AIT: mice sensitized with OVA, treated with OVA-AIT and challenged with OVA aerosol.

**Fig 1:**
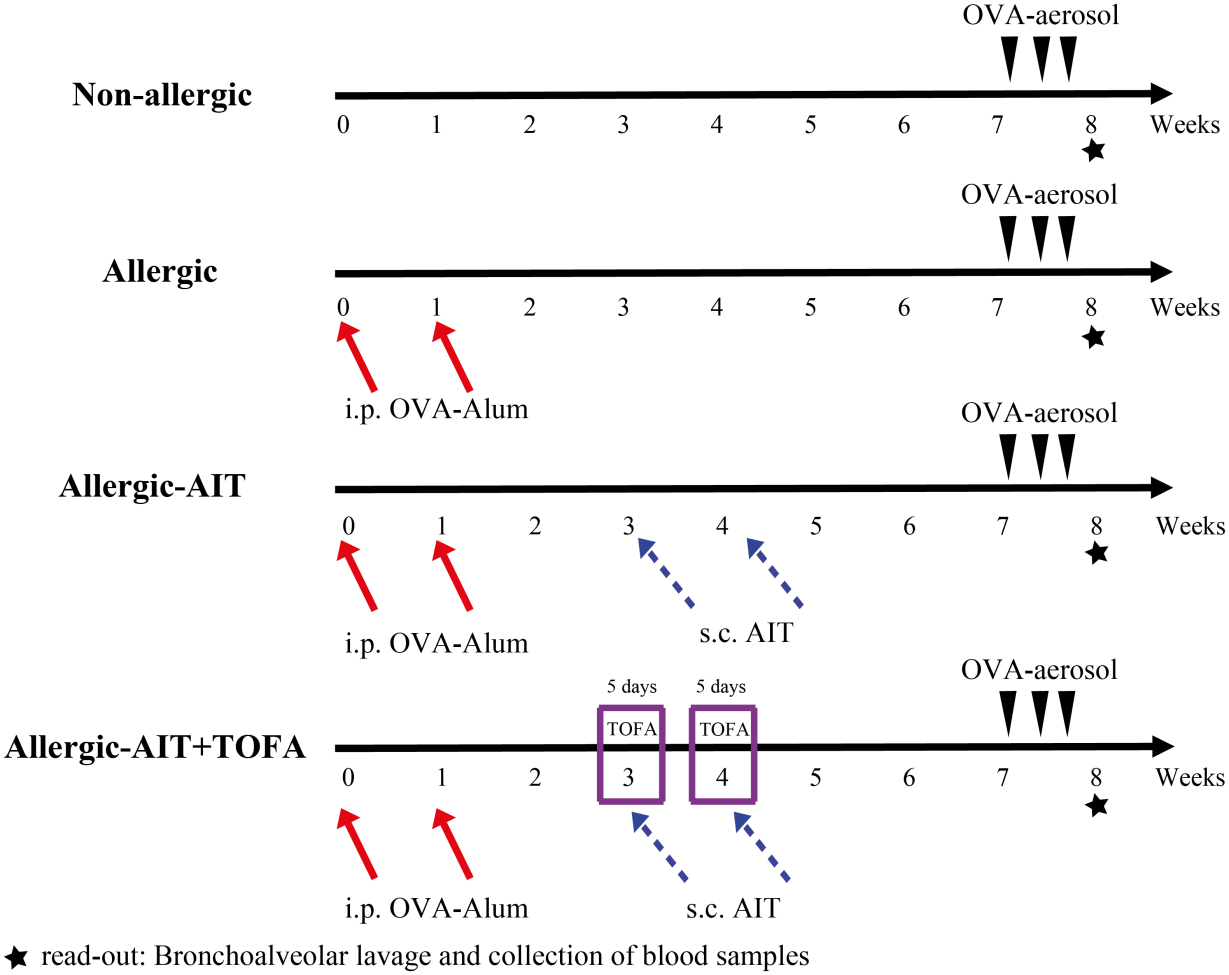
Schematic overview about the different *in vivo* treatment schemes (sensitization, AIT, TOFA administration, challenge). The treatment of four experimental groups of mice (n = 9–14) is shown. AIT, allergen-specific immunotherapy; i.p, intraperitoneal; s.c., subcutaneous.

Allergic-AIT + TOFA: mice sensitized with OVA, treated with OVA-AIT with concomitant gastric TOFA feeding and subsequent exposure to OVA-aerosol (Fig 1).

Allergic + TOFA: mice sensitized with OVA, treated with gastric TOFA feeding and subsequent exposure to OVA-aerosol.

### Measurement of antibodies in plasma

For the determination of total IgE levels, plasma samples and standards (mouse IgE clone C38-2; BD Pharmingen, Heidelberg, Germany) were transferred to microtiter plates, coated with 10 μg/mL monoclonal rat anti-mouse-IgE (IgG, clone-PC 284; The Binding Site, Schwetzingen, Germany). For OVA-specific IgE measurement, plasma samples and standards (mouse IgE anti-Ovalbumin clone: 2C6; Biozol, Eching, Germany) were transferred to microtiter plates coated with 2 μg/mL of OVA (chicken ovalbumin, Sigma, Germany). As secondary antibody, 2.5 μg/mL of biotinylated monoclonal rat anti-mouse IgE (clone R35-118; BD Pharmingen) were used, followed by incubation with BD OptEIA Reagent Set B (BD Pharmingen). Signal intensities were measured at 450 nm. The determination of further immunoglobulin isotype levels was performed with a combined electrochemiluminescence multiplexed assay system (Meso Scale Discovery, MSD, Rockville, MD USA).

### Measurement of cells in BAL fluid

Flow cytometry of BAL cells was performed by using the following monoclonal antibodies: anti-CD8a-AlexaFlour750 (clone 5H10, Thermo Fisher Scientific; AB_10374588), anti-Ly6c-FITC (clone AL-21, BD Biosciences, San Jose, CA USA; AB_394628), anti-CD4-PerCP-Cy5.5 (clone RM 4–5, BD Biosciences; AB_393977), anti-CD62L-PE-Cy7 (clone MEL-14, eBioscience, affymetrix, Frankfurt am Main, Germany; AB_469632), anti-CD3-eFlour450 (clone 17A2, eBiosciene; AB_1272229), anti-CD25-APC (clone PC61, BD Biosciences; AB_398623), anti-Gr1-PacificOrange (clone Rb6-8C5, Thermo Fisher Scientific; AB_2556571), anti-CD19-PE-Cy7 (clone 1D3, BD Biosciences; AB_10894021), anti-CD11b-APC-Cy7 (clone M1/70; BD Biosciences; AB_396772), anti-CD11c-FITC (clone HL 3, BD Biosciences; AB_395060), anti-CD45-AlexaFlour700 (clone 30-F11, BioLegend, San Diego, CA USA; AB_493714), anti-F4/80-APC (clone BM8, eBioscience; AB_469451) and anti-MHC-II (I-A/I-E)-PerCP-Cy5.5 (clone M5/114.15.2, BioLegend; AB_2191072) [23]. Data were acquired using a LSRII flow cytometer (BD Bioscience) and further analyzed with FACSDiva software (BD Biosciences) and FlowJo V.7.2.2 (Tree star, Ashland, USA). The gating strategy is displayed in S1 Fig.

### Histological analysis of the lung

After BAL, the lungs were removed and snap frozen in liquid nitrogen. For histological analysis, lungs were post-fixed in 4% buffered formaldehyde solution, dehydrated and embedded in paraffin. Four μm sections were stained with hematoxylin-eosin and periodic acid-Schiff (PAS).

### Cytokine measurements

Pro-inflammatory cytokines from BAL and plasma were analyzed by an electrochemiluminescence multiplex assay (Meso Scale Discovery, MSD). IL-13 and IL-17 were measured using the kits Mouse IL-13 CytoSet (Thermo Fisher Scientific) and Mouse IL-17A ELISA Max (BioLegend) according to manufacturer’s recommendations.

### Statistical analyses

Gaussian distribution was tested by D’Agostino & Pearson omnibus normality test. Gaussian and non-Gaussian distributed results were further analyzed by unpaired t test or Mann Whitney test, respectively (GraphPad Prism, San Diego, CA, USA). P-values of >0.05, ≤0.05, ≤0.01, ≤0.001 and ≤0.0001 are shown as ns, *, **, ***, and, ****, respectively.

### Human blood donors

For the T cell differentiation assay, naive T cells were isolated from the blood of 3 healthy donors (2 female, 1 male). The study was approved by the local ethics committee of the Technical University Munich, ethics board no 2877/10, and all individuals gave informed written consent.

### Differentiation of human naive CD4^+^ T cells into FOXP3^+^CD4^+^ T cells

Human peripheral blood mononuclear cells were isolated from heparinized whole blood by standard density gradient centrifugation (Lymphoprep, Axis Shield, Oslo, Norway). Naive CD4^+^ T cells were isolated using the Naive CD4^+^ T cell Kit II (Miltenyi Biotec, Bergisch Gladbach, Germany) and by additional depletion of CD45RO^+^ cells (CD45RO microbeads, Miltenyi Biotec). Cells were cultured in RPMI 1640 (Thermo Fisher Scientific) complete (10% FCS, Biochrom, Merck, Berlin, Germany; 2mM L-glutamine, 100 U/mL Penicillin/Streptomycin, Thermo Fisher Scientific) in 24-well plates at a concentration of 1x10^6^ cells/mL at 37°C. After isolation, cells were stimulated with plate-bound anti-CD3 (10 μg/mL; clone UCHT1, monoclonal, BD Biosciences; AB_395736) and 2 μg/mL anti-CD28 (clone CD28.2, monoclonal, BD Biosciences, 2 μg/mL; AB_396068) in solution and cultured for five days. To induce FOXP3 expression, the culture medium was supplemented with 50 U/mL rIL-2 (Novartis, Nürnberg, Germany) and 5 ng/mL TGF-β1 (Promokine). Beneath this control, cells were cultured in the presence of different concentrations of TOFA, Rapamycin (Sigma-Aldrich) and Cyclosporine A (Sigma-Aldrich). After three days of culture, half of the medium was removed and replaced by fresh RPMI 1640 complete, supplemented with the same doses of cytokines and agents as at day 0.

After five days of culture, the cells were washed with ice-cold PBS and stained with LIVE/DEAD Fixable Aqua Dead Cell Stain Kit (Thermo Fisher Scientific) according to the manufacturer’s protocol. Moreover the cells were stained 1:300 with anti-CD4-Alexa Flour700 (clone RPA-T4, monoclonal, BioLegend; AB_493743) and 1:100 with anti-FOXP3-eFlour450 (clone PCH101, monoclonal, eBioscience; AB_1834365) by using the FOXP3/Transcription Factor Staining Buffer Set (eBioscience, affymetrix) according to manufacturer’s protocol. The acquisition of cells was performed with BD FACSDIVA 7.0 on a BD LSR Fortessa (BD Biosciences). Data were analyzed with the software FlowJo (Tree Star, Ashland, OR), the lymphocyte population was gated on CD4^+^ cells and dead cells were excluded from the analysis.

## Results

### Effect of AIT and JAK inhibition on lung cell infiltration

**Fig 2:**
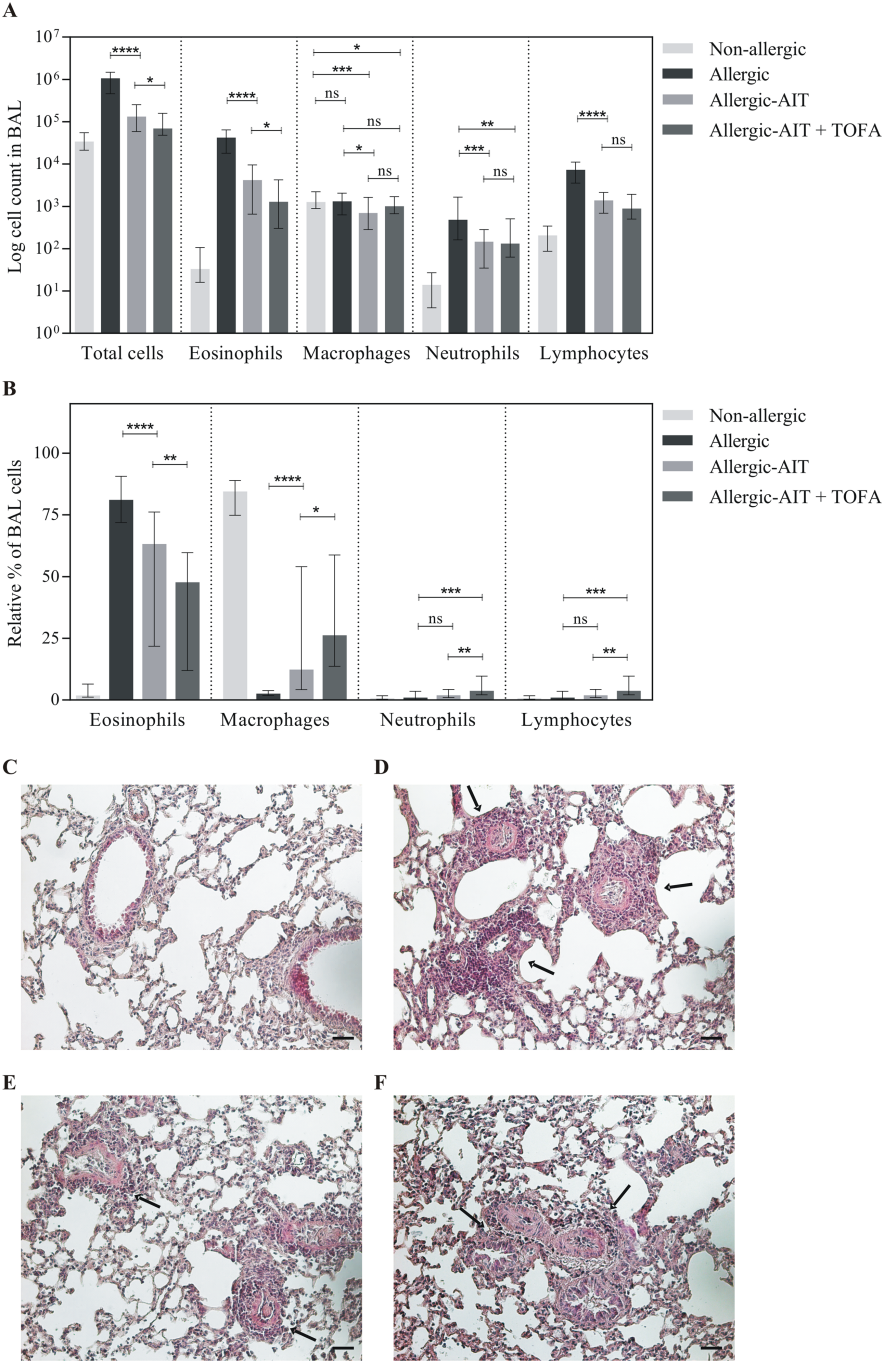
Analysis of the cellular BAL fluid compartment and lung histology of mice (Non-allergic, Allergic, Allergic-AIT, Allergic-AIT+TOFA) after OVA-aerosol challenge. (A) Counts of total BAL cells and total cell count of eosinophils, macrophages, neutrophils and lymphocytes. (B) Relative percentages of eosinophils, macrophages, neutrophils and lymphocytes within the total BAL cells. Shown is the median with range. Gaussian and non-Gaussian distributed results were analyzed by unpaired t test or Mann Whitney test, respectively. (C-F): Lung histology (H&E staining). (C) Non-allergic. (D) Allergic. (E) Allergic-AIT. (F) Allergic-AIT + TOFA. Arrows indicate inflammatory infiltrates; scale bar: 50 μm.

Sensitization and challenge with OVA (Fig 1) resulted in a massive cell infiltrate (median 31-fold compared to the non-allergic/OVA-challenged mice) in the BAL fluid, which was dominated by eosinophils (1279-fold) (Fig 2). AIT ameliorated OVA-induced total BAL cell infiltration in allergic-AIT mice by 87% compared to allergic mice. The combination treatment with AIT and TOFA led to further significant suppression of the BAL infiltrate to 93% (Fig 2A).

Moreover, AIT improved OVA-induced eosinophil BAL cell infiltration to 63% (median) compared to 81% in allergic mice. An additional significant reduction of eosinophil infiltration to approximately 48% was achieved by co-administration of TOFA (Fig 2B). This reduction was accompanied by a significant relative increase of macrophages, neutrophils and lymphocytes.

In contrast to non-allergic mice (Fig 2C) strong perivascular, peribronchiolar and alveolar inflammatory cell infiltration characterized by a strong eosinophilia was observed in the lungs of allergic mice (Fig 2D) and this effect was substantially reduced by AIT (Fig 2E) and even further by the combination of AIT and TOFA (Fig 2F). However, no reduction in PAS positive material was seen following AIT and AIT in combination with TOFA (data not shown).

**Fig 3:**
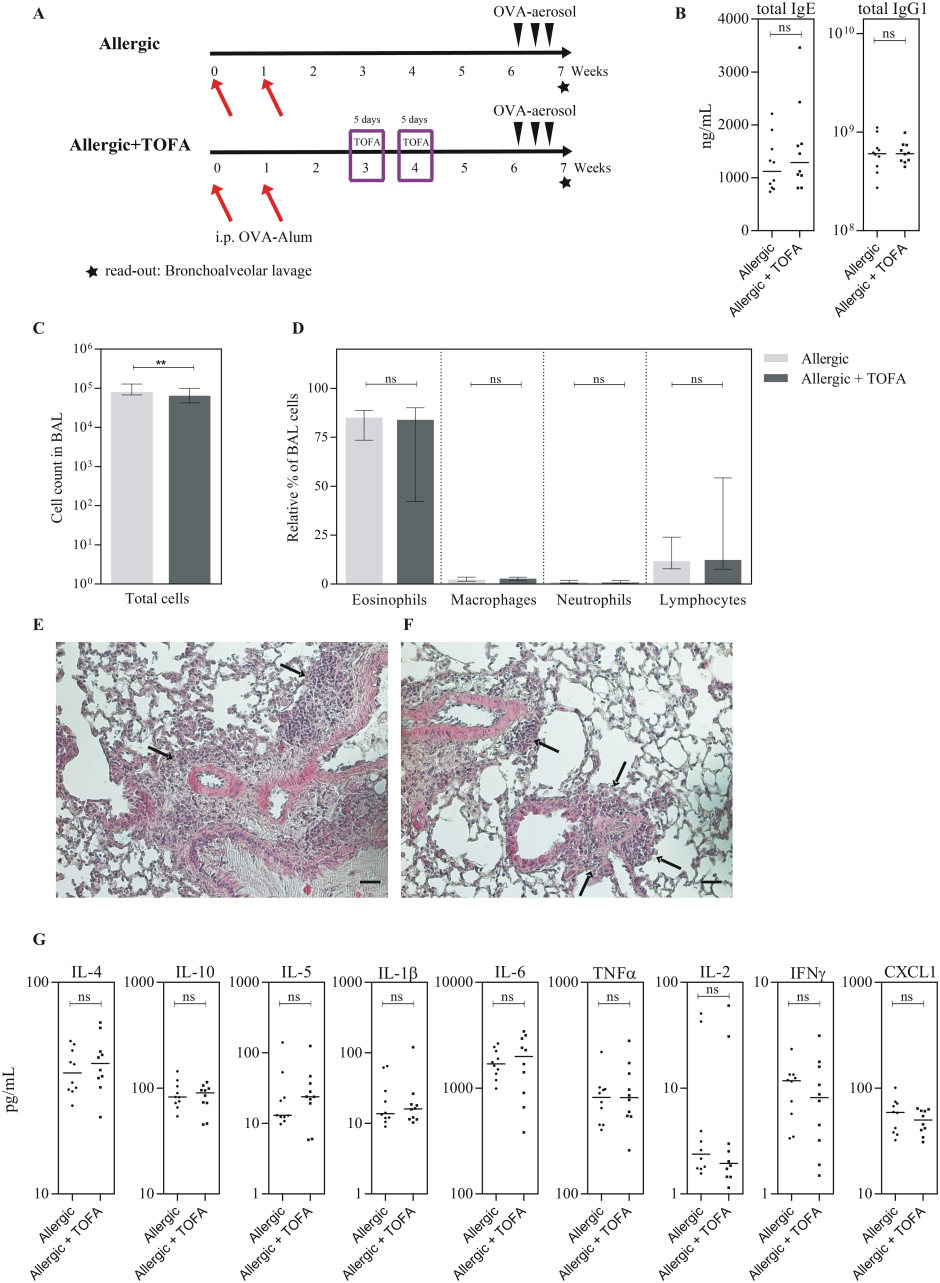
Effect of TOFA on allergic sensitization. (A) Schematic overview about the different *in vivo* treatment schemes (sensitization, TOFA administration, challenge) for two experimental groups of mice (n = 10). i.p, intraperitoneal. (B) Immunoglobulin measurements in plasma samples. (C) Counts of total BAL cells. (D) Relative percentages of eosinophils, macrophages, neutrophils and lymphocytes within the total BAL cells. Shown is the median with range. (E-F) Lung histology (H&E staining). (E) Allergic. (F) Allergic + TOFA. Arrows indicate inflammatory infiltrates; scale bar: 50 μm. (G) Results of cytokine measurements in the BAL fluid. Bars indicate the median. Gaussian and non-Gaussian distributed results were analyzed by unpaired t test or Mann Whitney test, respectively.

Of note, TOFA treatment without AIT was not able to induce any of these effects, verifying that no transient immunosuppression was responsible for the obtained results (Fig 3). Here, analyses were performed in week 6 to assess the time of complete wash-out of TOFA and to avoid transient TOFA mediated effects on experimental AIT (Fig 3A). In detail, TOFA treatment alone had no effect on immunoglobulin levels in plasma (Fig 3B), only slightly reduced the total cell number in the BAL fluid (Fig 3C), but had no effect on eosinophilic infiltration into the BAL fluid (Fig 3D) or on inflammatory cell infiltration in lung tissue (Fig 3E–3F). Moreover, no effects of TOFA-treatment without AIT on cytokine levels in the BAL fluid were observed (Fig 3G).

### Effect of AIT and JAK inhibition on cytokine secretion in BAL fluid

**Fig 4:**
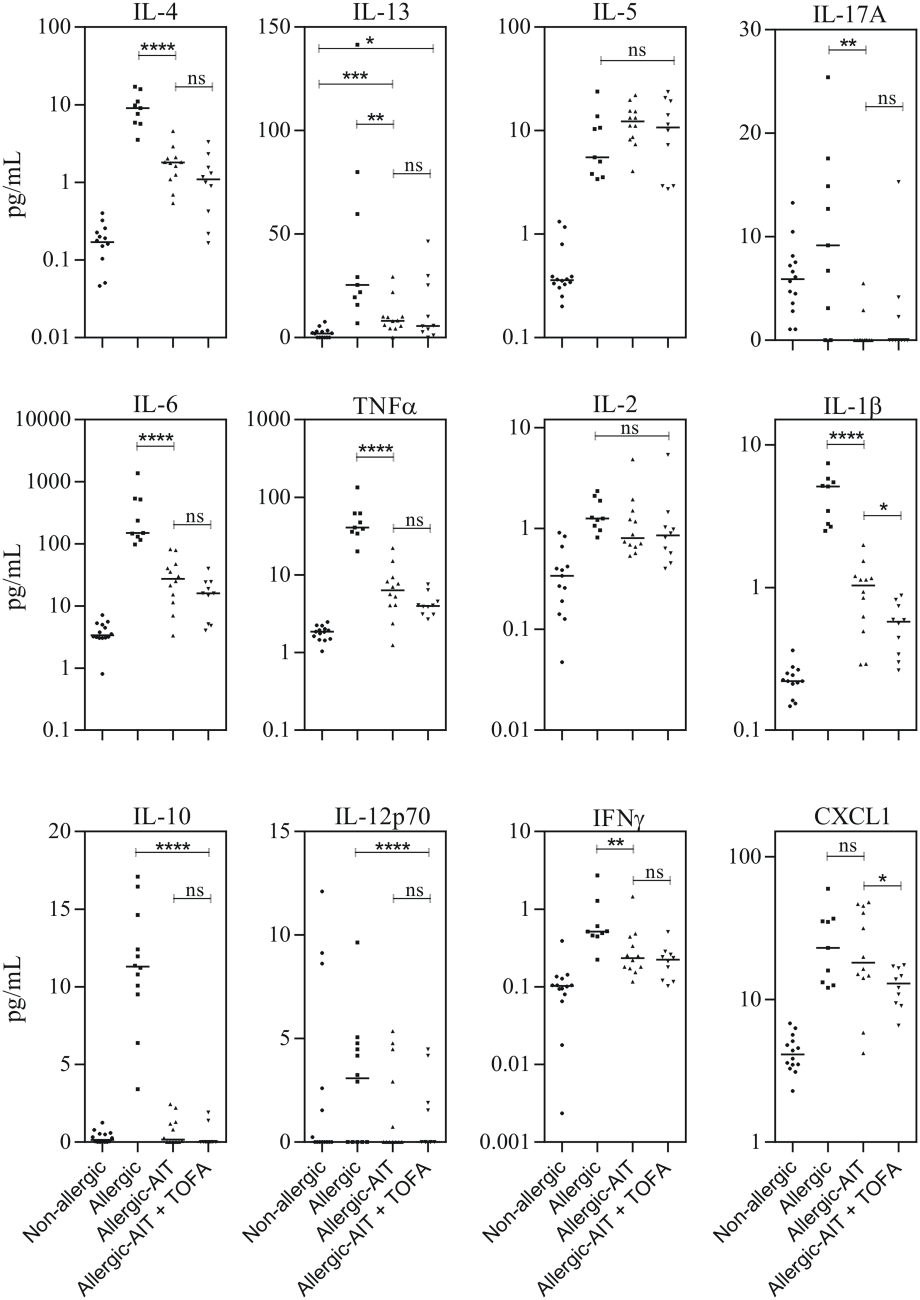
Analysis of the soluble BAL fluid compartment (Non-allergic, Allergic, Allergic-AIT, Allergic-AIT+TOFA) after OVA-aerosol challenge. Results of the cytokine measurement for IL-4, IL-13, IL-5, IL-17A, IL-6, TNF-α, IL-2, IL-1β, IL-10, IL-12p70, IFN-γ and CXCL1. Bars indicate the median. Gaussian and non-Gaussian distributed results were analyzed by unpaired t test or Mann Whitney test, respectively.

After OVA challenge the amount of several cytokines in the BAL fluid was assessed. Intriguingly, AIT induced a dramatic reduction of monokine secretion (IL-1β, IL-6 and TNF-α) into the BAL fluid (reduction to 20% of Il-1β, 18% of IL-6 and 16% of TNF-α compared to allergic mice) (Fig 4). The combination of AIT plus TOFA was even more potent (reduction of both Il-1β and IL-6 levels to 11%, and TNF-α levels to 10% compared to allergic mice). The addition of TOFA to AIT led to significantly reduced IL-1β secretion compared to AIT alone, while a tendency was observed for further reduction of IL-6 and TNF-α secretion.

A similar enhancement of inhibition by AIT and the combination of AIT and TOFA (median 78% and 56% compared to allergic mice) was observed for the chemokine CXCL1, the murine homologue of human IL-8, whereby only the addition of TOFA led to a significant reduction (Fig 4). In contrast, T cell cytokines IL-2 and IFN-γ were barely detectable and marginally reduced by AIT without further effect by concomitant TOFA treatment. While OVA challenge resulted in measurable amounts of IL-17A in non-allergic and allergic mice, this cytokine was not detectable in most mice after treatment with AIT or the combination of AIT and TOFA.

AIT significantly reduced the secretion of the Th2-cytokines IL-4 and IL-13 into BAL fluid and a tendency towards further decrease by the combination of AIT and TOFA was observed. In addition, the level of IL-10 in the BAL fluid was significantly decreased by AIT ant the combination of AIT and TOFA. However, both treatment regimens slightly increased IL-5 secretion (Fig 4).

### Effect of AIT and JAK inhibition on antibody levels

**Fig 5:**
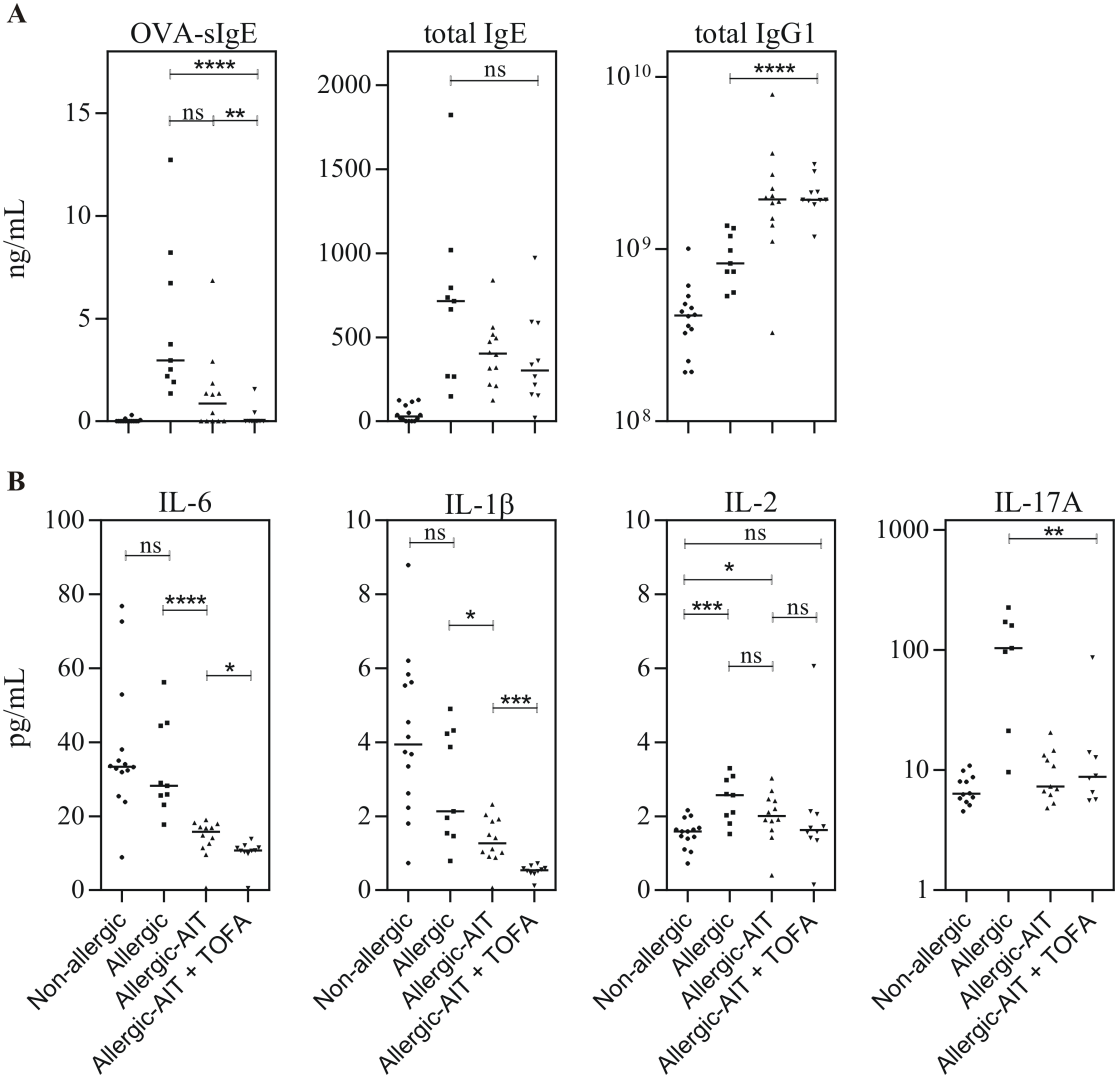
Analysis of murine plasma samples (Non-allergic, Allergic, Allergic-AIT, Allergic-AIT+TOFA) after OVA-aerosol challenge. (A) Results of the immunoglobulin measurement for OVA-specific IgE, total IgE and total IgG1, and (B) results of the cytokine measurement for IL-6, IL-1β, IL-2 and IL-17A. Bars indicate the median. Gaussian and non-Gaussian distributed results were analyzed by unpaired t test or Mann Whitney test, respectively.

Sensitization of mice with OVA resulted in the induction of OVA-specific IgE (Fig 5A, left panel). While only a tendency towards reduced sIgE was achieved by AIT alone, the combination treatment of AIT and TOFA resulted in a highly significant decrease of the amount of sIgE, which was comparable to that of non-allergic mice. Moreover, treatment of mice with AIT and the combination of AIT and TOFA led to an increasing tendency of reduced total IgE levels (median 56.5% and 42.2% compared to allergic mice (Fig 5A, middle panel). Additionally, AIT as well as the combination of AIT and TOFA resulted in a highly significant increase of total IgG1 levels compared to the allergic mice (Fig 5A, right panel). The levels of IgM, IgA, IgG2b and IgG3 were not affected by AIT or the combination of AIT and TOFA (data not shown).

### Effect of AIT and JAK inhibition on systemic cytokine levels

Non-allergic and allergic mice showed comparable levels of IL-6 in plasma after OVA-aerosol challenge. Interestingly, a reduction of the IL-6 plasma level was detected in mice after AIT (median 56% compared to allergic mice) with further significant reduction in mice treated with AIT and TOFA (median 38% compared to allergic mice) (Fig 5B). A similar enhancement of inhibition (41% and 75%) was observed for IL-1β. While AIT was able to reduce the IL-1β level to 60% compared to allergic mice, the combination of AIT and TOFA further decreased the level to 25%. Additionally, the co-administration of AIT and TOFA, in contrast to AIT alone, was able to reduce the level of IL-2 to the level of non-allergic mice. Interestingly, the level of IL-17A was only increased in allergic mice while it was comparable in non-allergic mice and mice treated with AIT or the combination of AIT and TOFA. IL-4, IL-13, IL-5, TNF-α, INF-γ, CXCL1 and IL-10 were barely detectable in plasma or showed only non-significant differences between different groups (S2 Fig).

### Tofacitinib facilitates the differentiation of human FOXP3^+^CD4^+^ T cells

**Fig 6:**
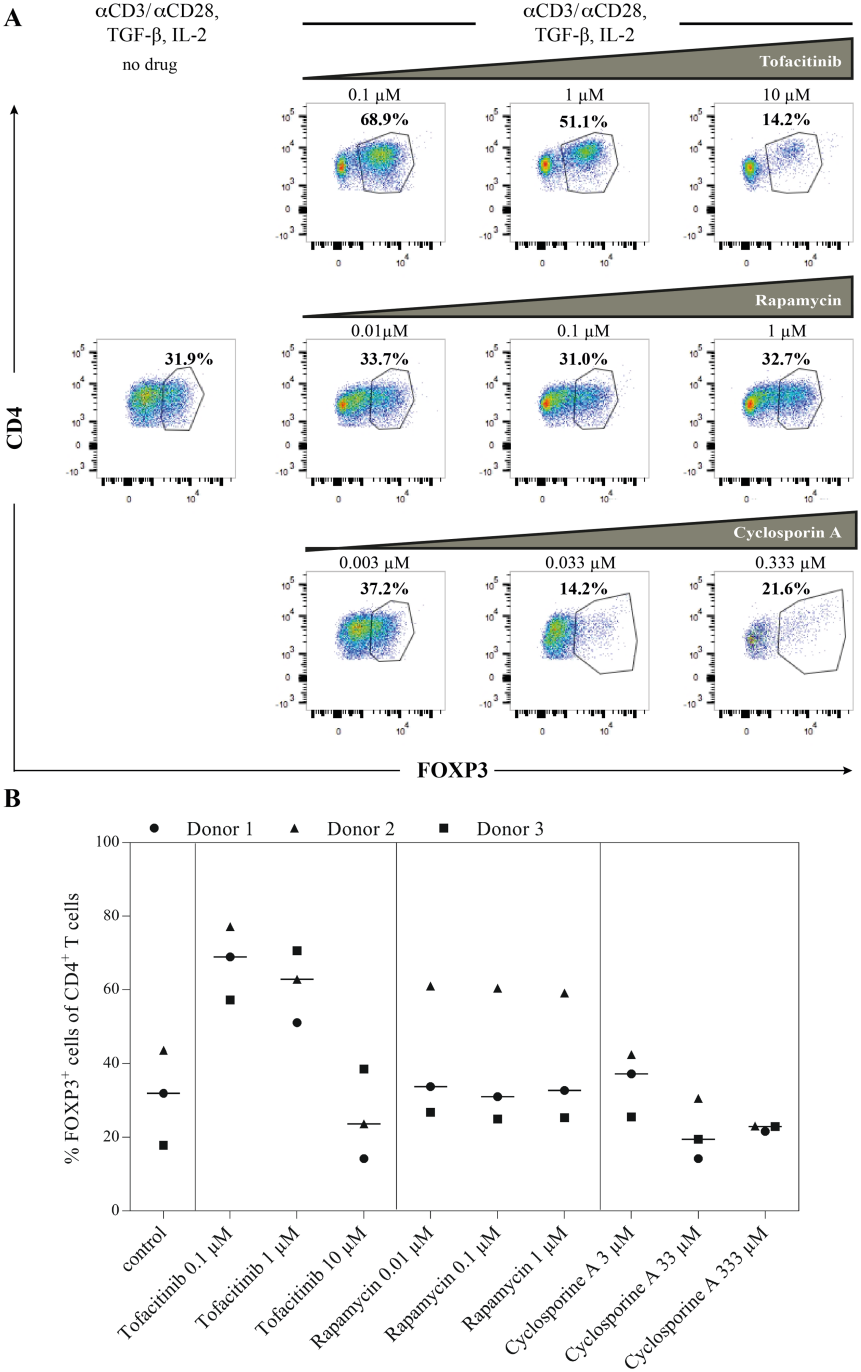
Effect of TOFA on the differentiation of human FOXP3^+^CD4^+^ T cells. Naive CD4^+^ T cells of human blood donors (n = 3) were stimulated with anti-CD3/anti-CD28, IL-2 and TGF-β1 to induce FOXP3 expression. In addition to this polarization control, TOFA, Rapamycin or Cycloysporine A were added to the medium in different concentrations. (A) Flow cytometry plots of anti-CD4 and anti-FOXP3 staining are shown for one representative donor. (B) The percentages of FOXP3^+^ cells within the living CD4^+^ T cell population under control, TOFA, Rapamycin and Cyclosporine A condition is given for the three donors. Bars indicate the median.

Since the suppressive effect of TOFA on human and murine Th1/Th2/Th17 effector cells and immunoglobulin secretion has been shown previously [18], we analyzed the Treg-induction as important immune tolerance mechanism. In order to obtain insights into the effect of TOFA and other immune-modulating agents such as Rapamycin and Cyclosporine A on FOXP3 expression in human CD4^+^ T cells, naive CD4^+^ T cells (purity 95–98%, data not shown) were isolated from healthy human blood donors (n = 3) and cultured in the presence of TGF-β1, IL-2 and different concentrations of the immune modulators. For the control cells, cultured under iTreg (induced regulatory T cell)-polarizing conditions without immune modulators, 17.7–43.6% FOXP3^+^ cells within the living CD4^+^ T cell population were detected (Fig 6). The two lower doses of TOFA were able to enhance the induction of FOXP3 expression within this T cell compartment (Fig 6). For a concentration of 0.1 μM and 1 μM TOFA in the culture medium 57.2–77.1% and 51.1–70.6% FOXP3 producing cells were detected, respectively. On average 0.1 μM and 1 μM TOFA were able to increase the percentage of FOXP3 expressing cells about 2.4 (+/- 0.7) − 2.5 (+/-1.3)-fold. The concentration of 10 μM of TOFA induced cytotoxicity (data not shown) and diminished the FOXP3-enhancing effect. Rapamycin treatment did not inhibit FOXP3 expression and resulted in 1.3-fold higher expression. The lowest concentration of Cyclosporine A (0.003 μM) showed no effect on FOXP3 induction, whereas the two higher concentrations (0.033 μM, 0.333 μM) reduced the percentage of FOXP3^+^ cells within the CD4^+^ T cell compartment. The obtained results were consistent for all individual donors (Fig 6B).

## Discussion

In order to identify immune modulators which have the potential to improve the efficacy of allergen-specific immunotherapy we addressed the beneficial effect of Tofacitinib during AIT. The rationale behind this combinatorial approach is that vaccination-induced antigen presentation in the absence of inflammatory signals might facilitate the induction of immunological tolerance [26]. So far, the effects of the application of JAK inhibitors during AIT have not been reported.

In this study we used a murine model of OVA-induced allergy and subsequent experimental AIT, similar to the model previously described by Vissers et al. [27]. In our model the application of TOFA in combination with AIT was able to improve several key parameters of allergic airway inflammation, such as total cell and particularly eosinophilic infiltration into the BAL fluid, levels of the Th2 cytokines IL-4 and IL-13, the monokines IL-1β, IL-6 and TNF-α as well as IL-17A and CXCL1. Moreover, OVA-specific as well as total IgE levels were clearly reduced. Particularly sIgE levels could only be reduced with high significance by the co-administration of AIT and TOFA. Moreover, a systemic inhibition of pro-inflammatory cytokines was observed. Hence, the inhibition of the JAK 1 and 3 pathways demonstrates clear potential for further improvement of specific immunotherapy. Although TOFA shows the highest selectivity for JAK1 and 3, effects by the weaker inhibition of JAK 2 and TYK2 are known [28]. However, we could show that the improved efficacy of AIT mediated by TOFA is not primarily due to an inhibition of cellular immunologic effector functions, but also by the functional and robust induction of potentially protective IgG1 antibodies. Furthermore, comparable effects on airway eosinophilia and Th2 cytokine production were previously described for the administration of the highly selective JAK1/3 inhibitor R256 during the sensitization and allergen challenge phase [29].

Key problems of human AIT are the side effects, such as local swelling, granuloma formation, anaphylaxis, as well as often insufficient clinical responses, which are related to local inflammatory conditions [4]. Therefore, the aim of short-term administration of TOFA during AIT is not a long-lasting modification of the JAK/STAT pathway but rather a short-term blockade of this signaling cascade to restrict local inflammation and to support the tolerogenic memory effect induced by AIT. This study demonstrates that TOFA-mediated JAK inhibition can significantly support AIT-mediated control of local inflammation, in particular the Th2 dependent influx of eosinophils.

Although the analysis of clinical trials is too multifactorial and requires large sample sizes, it can be anticipated that eosinophil reduction positively relates to clinical efficacy [12]. The TOFA-mediated downregulation of eosinophil infiltration is not related to Th2 cytokines, in particular IL-5, which was not decreased by AIT or AIT in combination with TOFA. Interestingly, IL-4 and IL-13 were downregulated by AIT and the combination of AIT and TOFA. Since the genes of IL-4 and IL-5 are situated on the same genomic locus [30], it is unlikely that the differential secretion levels originate from the same cells, but rather are a result of different cell origin such as Th2 cells and innate lymphoid type 2 cells [31]. In addition the level of IL-10 in the BAL fluid, which is also produced by Th2 cells [32], was downregulated by AIT and the combination of AIT and TOFA. Unfortunately, our study lacks lung function measurements, which would enhance its clinical relevance.

The induction of IgG1 by AIT is in line with previous studies, which have shown that AIT with OVA in murine allergic airway disease induces OVA-specific IgG1 [33]. It is of major importance that this induction of potentially protective antibodies is not affected by TOFA administration. Recently, a study focusing on the effects of TOFA treatment on human B cells showed results broadening our findings for the human situation. Rizzi et al demonstrated that *in vitro* TOFA treatment does not inhibit class switching during B cell maturation and only diminishes plasmablast formation and immunoglobulin secretion in naive B cells for a short period. B cells isolated from TOFA-treated patients could be again activated to differentiate into plasmablasts and these plasmablasts secreted immunoglobulin levels comparable or slightly increased to normal levels [34]. We might speculate that during the short-term TOFA treatment combined to AIT, we inhibit the prompt formation of IgE producing plasmablasts and create a window of opportunity for the class switching to IgG. Allergen-IgG complexes can then further induce IgG1 and the secretion of macrophage-derived IL-10, which is an important mediator of immune tolerance in experimental murine AIT [35, 36]. These IgG1 antibodies are believed to compete with IgE in a similar way as IgG4 in the human system, what in turn prevents mediator release from sensitized mast cells [9, 10]. However, the reduction of the Th2-late phase response with eosinophil infiltration in the BAL-fluid is independent of B cell function and IgG-production [33].

The effect of TOFA-enhanced AIT was most pronounced among monokines IL-1β, TNF-α and IL-6. The mean terminal plasma half-life of TOFA is 3.2 hours [37] and it has been previously shown that discontinuation of TOFA after short-term treatment *in vitro* allows normal reactivation of lymphocytes within four days [38]. Therefore, the effect at the time point of aerosol allergen challenge cannot be attributed to the transient action of TOFA, since the last application of TOFA was given 18 days before. The reduction of IL-6 has also been observed in a four weeks clinical trial assessing TOFA for rheumatoid arthritis [39]. Taken together, it is likely that resident epithelial cells (e.g. bronchial/ alveolar or liver epithelial cells) mediate the reduction of pro-inflammatory mediators, as it was shown in a recent model of graft-versus host disease for keratinocyte-derived CXCL9 and CXCL10 [40]. It is currently unknown, whether the TOFA-mediated reduction of IL-1β responses are caused by a long-lasting effect on airway- or liver epithelial cells or by AIT-modulated T cell responses affecting epithelium-regulating cytokines.

Interestingly, we were able to demonstrate that TOFA administration *in vitro* favored the induction of human FOXP3^+^CD4^+^ T cells, a hallmark of T cell tolerance [12]. This extends the findings of an earlier study, which demonstrated that TOFA preserves the function of regulatory T cells and inhibits effector T cells [18]. In contrast, other immunosuppressive drugs such as Cyclosporine A inhibit FOXP3-mediated Treg induction [15]. We previously demonstrated that this effect depends on direct inhibition of NFAT (nuclear factor of activated T cells) binding to the proximal FOXP3 promoter [41].

Our data demonstrate that JAK-inhibitors are able to shift the balance of newly induced T cell reactivity. This potentially favors the induction of functional regulatory T cells, which build the basis for a long-lasting modulation of the immunological memory and which are beneficial for the treatment of a variety of immune diseases. Additionally, this hypothesis is supported by a recent study of a murine autoimmune disease model, showing the antigen-specific induction of Tregs and a decrease of Th1 and Th17 by dendritic cells treated with TOFA *in vitro* [42].

## Conclusion

Taken together the current study shows that the combination treatment of AIT with a JAK inhibitor amplifies the clinical efficacy of AIT, as it not only influences adaptive, but also the innate arm of immune responses. The promising preclinical data on the long-lasting effects on local and systemic inflammation pave the way towards the set-up of human clinical trials. These trials could open up the chance to further minimize local and systemic side effects, even during seasonal treatment or in polysensitized patients suffering from perennial symptoms, using uncritical short-term treatment [21] with a JAK inhibitor.

## Supporting information

S1 Fig Analysis of cells in BAL fluid by flow cytometry. The FlowJo_V10 software was used to select BAL fluid cells on 2-D plots. First, live (7AAD negative) CD45+ leukocytes were selected to eliminate cell debris, erythrocytes and dead cells. Then lymphocytes were gated on SSC-A^low^/CD11b- to further discriminate CD19+ B cells and CD3+ T cells. Neutrophils were identified as SSC-A^low^/GR-1+. Pre-gating on SSC-A^mid-high^/GR-1- cells allowed to discriminate eosinophils from macrophages by further staining of MHC-II and CD11c.
(TIF)

S2 Fig Analyses of murine plasma samples (Non-allergic, Allergic, Allergic-AIT, Allergic-AIT+TOFA) after OVA-aerosol challenge. Results of the cytokine measurement for IL-4, IL-13, IL-5, TNF-α, IFN-γ, CXCL1 and IL-10. Regarding the measurement of IL-13, a statistical analysis was not performed, because in most of the samples IL-13 was under the detection limit. Bars indicate the median. Gaussian and non-Gaussian distributed results were analyzed by unpaired t test or Mann Whitney test, respectively.
(TIF)
