# Supplementary figures and images for: Improved efficacy of allergen-specific immunotherapy by JAK inhibition in a murine model of allergic asthma
Source: PLoS One. 2017 Jun 1;12(6):e0178563. doi: 10.1371/journal.pone.0178563 (PMC5453633; doi:10.1371/journal.pone.0178563)

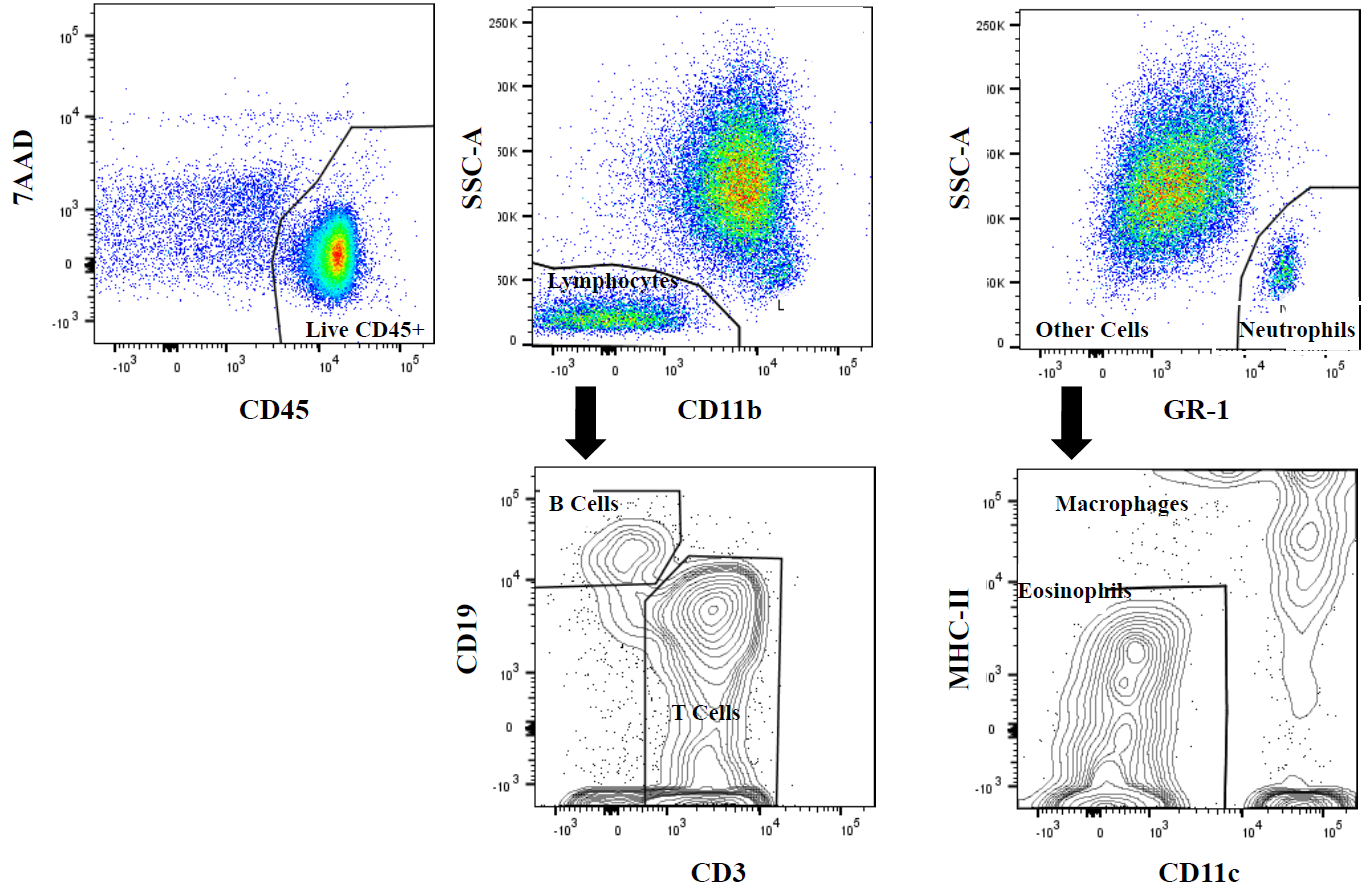

Supplement: S1 Fig — The FlowJo_V10 software was used to select BAL fluid cells on 2-D plots. First, live (7AAD negative) CD45+ leukocytes were selected to eliminate cell debris, erythrocytes and dead cells. Then lymphocytes were gated on SSC-Alow/CD11b- to further discriminate CD19+ B cells and CD3+ T cells. Neutrophils were identified as SSC-Alow/GR-1+. Pre-gating on SSC-Amid-high/GR-1- cells allowed to discriminate eosinophils from macrophages by further staining of MHC-II and CD11c. (TIF) [file pone.0178563.s001.tif]

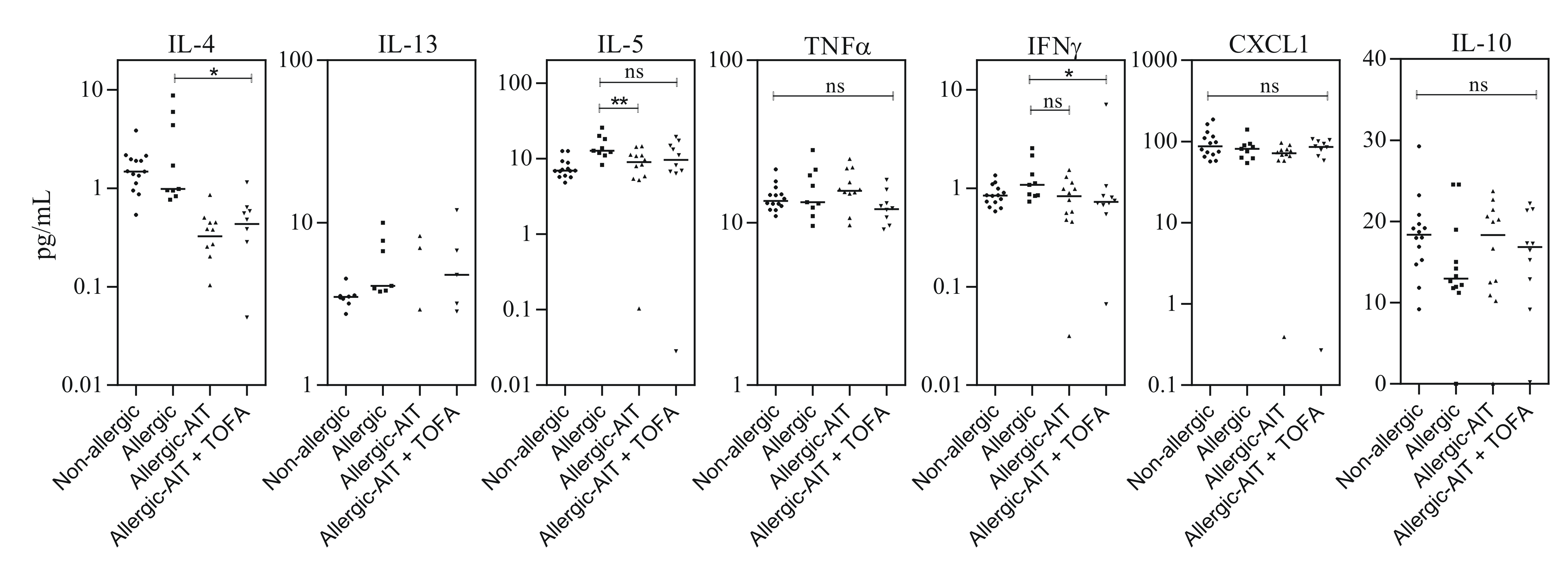

Supplement: S2 Fig — Results of the cytokine measurement for IL-4, IL-13, IL-5, TNF-α, IFN-γ, CXCL1 and IL-10. Regarding the measurement of IL-13, a statistical analysis was not performed, because in most of the samples IL-13 was under the detection limit. Bars indicate the median. Gaussian and non-Gaussian distributed results were analyzed by unpaired t test or Mann Whitney test, respectively. (TIF) [file pone.0178563.s002.tif]
